# Rationale, design, and profile of Comprehensive Registry of In-Hospital Intensive Care for OHCA Survival (CRITICAL) study in Osaka, Japan

**DOI:** 10.1186/s40560-016-0128-5

**Published:** 2016-01-26

**Authors:** Tomoki Yamada, Tetsuhisa Kitamura, Koichi Hayakawa, Kazuhisa Yoshiya, Taro Irisawa, Yoshio Abe, Megumi Ishiro, Toshifumi Uejima, Yasuo Ohishi, Kazuhisa Kaneda, Takeyuki Kiguchi, Masashi Kishi, Masafumi Kishimoto, Shota Nakao, Tetsuro Nishimura, Yasuyuki Hayashi, Takaya Morooka, Junichi Izawa, Tomonari Shimamoto, Toshihiro Hatakeyama, Tasuku Matsuyama, Takashi Kawamura, Takeshi Shimazu, Taku Iwami

**Affiliations:** Department of Traumatology and Acute Critical Medicine, Osaka University Graduate School of Medicine, Suita, Japan; Emergency and Critical Care Medical Center, Osaka Police Hospital, Osaka, Japan; Division of Environmental Medicine and Population Sciences, Department of Social and Environmental Medicine, Graduate School of Medicine, Osaka University, Suita, Japan; Department of Emergency and Critical Care Medicine, Kansai Medical University, Takii Hospital, Moriguchi, Japan; Department of Emergency Medicine, Tane General Hospital, Osaka, Japan; Department of Critical Care Medicine, Osaka City University, Osaka, Japan; Department of Emergency and Critical Care Medicine, Kinki University School of Medicine, Osaka-Sayama, Japan; Osaka Mishima Emergency Critical Care Center, Takatsuki, Japan; Critical Care and Trauma Center, Osaka General Medical Center, Osaka, Japan; Osaka Prefectural Nakakawachi Medical Center of Acute Medicine, Higashi-Osaka, Japan; Senshu Trauma and Critical Care Center, Osaka, Japan; Traumatology and Critical Care Medical Center, National Hospital Organization Osaka National Hospital, Osaka, Japan; Senri Critical Care Medical Center, Saiseikai Senri Hospital, Suita, Japan; Emergency and Critical Care Medical Center, Osaka City General Hospital, Osaka, Japan; Kyoto University Health Service, Yoshida-Honmachi, Sakyo-ku, Kyoto, 606-8501 Japan

**Keywords:** Out-of-hospital cardiac arrest, Outcome, In-hospital intensive care, Cohort, CRITICAL

## Abstract

**Background:**

We established a multi-center, prospective cohort that could provide appropriate therapeutic strategies such as criteria for the introduction and the effectiveness of in-hospital advanced treatments, including percutaneous coronary intervention (PCI), target temperature management, and extracorporeal cardiopulmonary resuscitation (ECPR) for out-of-hospital cardiac arrest (OHCA) patients.

**Methods:**

In Osaka Prefecture, Japan, we registered all consecutive patients who were suffering from an OHCA for whom resuscitation was attempted and who were then transported to institutions participating in this registry since July 1, 2012. A total of 11 critical care medical centers and one hospital with an emergency care department participated in this registry. The primary outcome was neurological status after OHCA, defined as cerebral performance category (CPC) scale.

**Results:**

A total of 688 OHCA patients were documented between July 2012 and December 2012. Of them, 657 were eligible for our analysis. Patients’ average age was 66.2 years old, and male patients accounted for 66.2 %. The proportion of OHCAs having a cardiac origin was 50.4 %. The proportion as first documented rhythm of ventricular fibrillation/pulseless ventricular tachycardia was 11.6 %, pulseless electrical activity 23.4 %, and asystole 54.5 %. After hospital arrival, 10.5 % received defibrillation, 90.8 % tracheal intubation, 3.0 % ECPR, 3.5 % PCI, and 83.1 % adrenaline administration. The proportions of 90-day survival and CPC 1/2 at 90 days after OHCAs were 5.9 and 3.0 %, respectively.

**Conclusions:**

The Comprehensive Registry of In-hospital Intensive Care for OHCA Survival (CRITICAL) study will enroll over 2000 OHCA patients every year. It is still ongoing without a set termination date in order to provide valuable information regarding appropriate therapeutic strategies for OHCA patients (UMIN000007528).

## Background

Out-of-hospital cardiac arrest (OHCA) of cardiac origin is one of the leading causes of death in the industrialized world [1], with approximately 70,000 events occurring every year in Japan [2]. The Utstein Osaka Project, a prospective, population-based cohort, was launched in Osaka, Japan in May 1998 [3] and has been providing valuable findings including the effectiveness of chest compression-only cardiopulmonary resuscitation (CPR) by bystanders [4] and the importance of continuously improving the chain of survival at the community level [5]. However, survival after OHCAs is still low, only <10 % even among bystander-witnessed OHCA patients [2].

This better survival after OHCAs in Osaka is mainly due to improvement of the prehospital emergency medical service (EMS). For further improvement of outcomes after OHCAs, measurement and assessment of the quality of in-hospital intensive care after hospital arrival will also be required, especially for OHCA patients with post-cardiac arrest syndrome (PCAS). In addition, several countries and regions including Asia, Europe, and the USA have recently been launching large-scale OHCA registries [6–10] because of the great need for high-quality data collection that can be used for improving OHCA outcomes. Thus, obtaining comprehensive data for both out- and in-hospital OHCA treatments, and understanding the actual conditions that will lead to improved OHCA outcomes, is one of the most urgent issues in resuscitation science.

In order to improve the survival after OHCA by providing appropriate therapeutic strategies incorporating criteria such as the introduction and effectiveness of in-hospital advanced treatments including percutaneous coronary intervention (PCI) [11], target temperature management (TTM) [12–14], and extracorporeal cardiopulmonary resuscitation (ECPR) [15, 16] for OHCA patients, we established a multi-center, prospective cohort that focused on OHCA patients who were transported to critical care centers or hospitals with an emergency care department staffed by EMS personnel. Herein, we will describe the study design and the profiles of cohort patients. This study has been designated as the Comprehensive Registry of In-Hospital Intensive Care for OHCA Survival (the CRITICAL study) [17].

## Methods

### Population and settings

The target area of the CRITICAL study is Osaka Prefecture in Japan, which has an area of 1897 km^2^ with a residential population of 8,865,245 inhabitants as of 2010 [18]. Males make up 48.3 % of the population, 22.4 % of whom are ≥65 years old. Osaka included 535 hospitals (108,481 beds) in 2012 [19]. Of them, 276 include 15 critical care medical centers (CCMCs) that can accept emergency severely ill patients from ambulances, including OHCA patients [20]. In this study, 11 of 13 CCMCs and one non-CCMC with an emergency care department in Osaka participated. Approximately 7500 OHCAs occur in Osaka every year [2]. As many as 30 % of OHCA patients in Osaka were transported to CCMCs and treated [21]. Therefore, this registry is planning to enroll over 2000 OHCA patients every year and is ongoing with no set ending to the study period. The study was approved by the Ethics Committee of Osaka University and Kyoto University as the corresponding institution, and each hospital also approved the CRITICAL study protocol as necessary.

### Study patients

We registered all consecutive patients who were suffering from an OHCA and for whom resuscitation was attempted and who were then transported to participating institutions starting on July 1, 2012. This study excluded OHCA patients who did not receive CPR by physicians or those with a disagreement about our registry, either by family members or themselves. The requirement of giving individual informed consent for the reviews of patients’ outcomes was waived by the Personal Information Protection Law and the national research ethics guidelines of Japan. This study described baseline characteristics and outcomes of OHCA patients who were transported to participating institutions from July 1, 2012 through December 31, 2012.

### Emergency medical service organization and equipment in Osaka

Details of the EMS system in Osaka were described previously [4, 5]. The 119 emergency telephone number is accessible anywhere in Japan including Osaka, and on receipt of a 119 call, an emergency dispatch center sends the nearest available ambulance to the site. Emergency services are provided 24 h every day; the system is single-tiered in 32 stations and two-tiered in two stations. The latter uses medics followed by physicians. Each ambulance includes a three-person unit providing life support. Most highly trained EMS personnel are called emergency life-saving technicians. They are allowed to insert an i.v. line and an adjunct airway and to use a semi-automated external defibrillator for OHCA patients. Emergency life-saving technicians are permitted to provide shocks without consulting a physician, and specially trained emergency life-saving technicians are allowed to carry out tracheal intubation and to administer epinephrine for OHCA patients. All EMS providers carried out CPR, basically according to the 2010 Japanese CPR guidelines during this study period.

Prehospital resuscitation data were obtained from the All-Japan Utstein Registry of the Fire and Disaster Management Agency of Japan. Details of the registry were described in detail in our preceding paper [22]. Data were collected prospectively with the use of a data form based on the Utstein-style international guideline of reporting OHCA [23, 24]. Collected data included the following: witness status, bystander-initiated CPR, shocks by public-access automated external defibrillators (AEDs), dispatcher instructions, first documented rhythm, shocks by EMS personnel, advanced airway management, intravenous fluid, adrenalin administration, and resuscitation time course.

### Data collection and quality control

In this registry, we collected detailed information on OHCA patients after hospital arrival. Anonymized data were fed into the Web form by physicians or medical staff in cooperation with physicians in charge of the patient, were logically checked by the system, and were finally confirmed by the CRITICAL study working group. If the data form was incomplete, the working group returned it to the respective institution and the data were completed. In-hospital data were systemically merged with Utstein-style prehospital data gathered from the FDMA by the working group, by the use of three important items in both data: emergency call time, age, and gender. The CRITICAL study has the following three detailed in-hospital data:

1. Hospital information
  Each participating hospital needed to enter hospital information at the time of registration. The required information was as follows: the type of emergency department (CCMC or non-CCMC); total bed number; intensive care unit bed number; pediatric intensive care unit bed number; annual expected number of OHCA patients; number of physicians and nurses who treated an OHCA patient (daytime and nighttime duty); the type of physicians (yes or no) for OHCA treatments such as acute care physicians, intensive care physicians, anesthesiologists, cardiologists, and pediatricians; intensive care unit training facility for board-certified intensivists approved by the Japanese Society of Intensive Care Medicine (yes or no); use of end-tidal carbon dioxide monitor during cardiopulmonary arrest (yes or no); ECPR use for an OHCA patient (yes or no); having an ECPR protocol (yes or no); person who performed the ECPR priming (physician or clinical engineer); body temperature management for OHCA (available or not); and body temperature management protocol (yes or no) and details such as target (maintenance) temperature, duration of target (maintenance) temperature, rewarming target temperature, and duration of rewarming.
2. Baseline OHCA patient information
  Baseline patient information was collected for both OHCA patient identification and entry criteria confirmation. First, information on the emergency call time from bystanders and the hospital arrival time, along with OHCA patient’s sex and age, were included. Next, patients who met the following criteria were registered: (1) OHCA occurred in prehospital settings, (2) was resuscitated by EMS personnel and then transported to the participated institutions or (3) was defibrillated by bystanders and then transported to the institutions, and (4) was resuscitated by physicians after hospital arrival.
3. In-hospital data including treatments, arterial blood gases, laboratory data, and outcomes
  In-hospital data on OHCA patients after hospital arrival were prospectively collected using an original report form. The cause of arrest was defined as having cardiac (acute coronary syndrome, other heart disease, presumed cardiac cause) or non-cardiac (cerebrovascular diseases, respiratory diseases, malignant tumors, external causes including traffic injury, fall, hanging, drowning, asphyxia, drug overdose, or any other external cause, and sudden infant death syndrome [only for children]) causes [23, 24]. The category of presumed cardiac cause was a diagnosis by exclusion (i.e., the diagnosis was made when there was no evidence of a non-cardiac cause). Diagnoses of cardiac or non-cardiac origin were clinically made by the physician in charge. Other baseline information are as follows: time of departure of ambulance or helicopter with physicians, return of spontaneous resuscitation (ROSC) after hospital arrival (or after contact with physicians in ambulance or helicopter), and first documented rhythm after hospital arrival (or after contact with physicians in ambulance or helicopter).
  The reporting form also required actual detailed treatments for OHCA patients (e.g., defibrillation, tracheal intubation, ECPR, intra-aortic balloon pumping (IABP), cardioangiography (CAG), percutaneous coronary intervention, target temperature management, drug administration during cardiopulmonary arrest [adrenalin, amiodarone, nifekalant, lidocaine, atropine, magnesium, and vasopressin]), arterial blood gases measured initially at hospital arrival (pH, PaCO_2_ [mmHg], PaO_2_ [mmHg], HCO_3_ [mEq/l], base excess [mEq/l], lactate [mmol/l], glucose [mg/dl]), and laboratory data measured initially at hospital arrival (blood urea nitrogen [mg/dl], creatinine [mg/dl], total protein [g/dl], albumin [g/dl], sodium [mEq/l], potassium [mEq/l], and ammonia [μg/dl]).
  Outcome data were also prospectively collected and included as follows [25]: condition after hospital arrival (admitted to intensive care unit/ward or death at emergency department), 1 month and 90-day survival, and neurological status at 1 month and 90 days after OHCA occurrence by using the cerebral performance category (CPC) scale (category 1, good cerebral performance; category 2, moderate cerebral disability; category 3, severe cerebral disability; category 4, coma or vegetative state; category 5, death) or pediatric CPC scale (category 1, normal cerebral performance; category 2, mild cerebral disability; category 3, moderate cerebral disability; category 4, severe cerebral disability; category 5, coma or vegetative state; category 6, death) if the patient was aged ≤17 years old. Survivors were evaluated 1 month and 90 days after the event for a neurologic assessment by the physician in charge.

### Statistical analysis

The *χ*^2^ test and one-way analysis of variance were used to analyze statistical differences from the first documented rhythm at EMS arrival. All *p* values were two-sided, and those less than 0.05 were considered to be statistically significant. Data were shown as mean ± standard deviation and the percentage of which number. All statistical analyses were carried out using SPSS software (version 22J, IBM Corp., Armonk, NY).

## Results

**Fig. 1:**
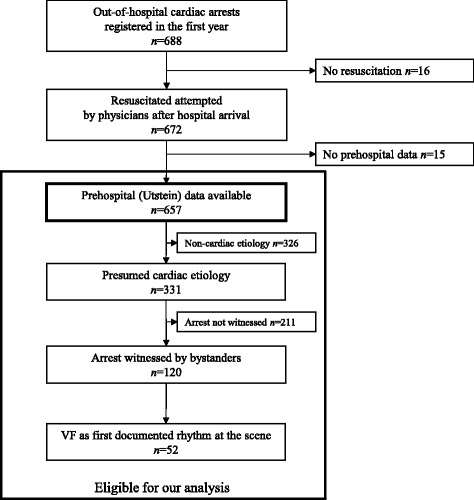
Patient flow

Figure 1 shows an overview of the study patients. A total of 688 OHCA patients were documented between July and December 2012. Excluding 16 patients who were not resuscitated by physicians after hospital arrival and 15 patients without prehospital data, 657 patients were eligible for our analysis. Of them, 52 were bystander-witnessed ventricular fibrillation (VF) arrests presumed to be of cardiac etiology.

Registered hospital characteristics are shown in Table 1. The institutions had an average of 570 beds, and the expected number of OHCA patients transported to each institution was 171 every year. All institutions had ≥3 physicians treating OHCAs during the day. Nine institutions had an ECPR protocol for OHCA treatments and ten institutions had a body temperature management protocol for them.

**Table 1:** Hospital characteristics

Institutions	(*n* = 12)	
Critical emergency medical center, *n* (%)	11	(91.7)
Total bed number, mean (SD)	570.2	(408.9)
Intensive care unit bed number, mean (SD)	13.1	(7.1)
Pediatric intensive care unit bed number, mean (SD)	0	(0)
Annual expected number of OHCA cases, mean (SD)	171.4	(72.1)
≥3 physicians treated an OHCA case (daytime duty), *n* (%)	12	(100)
≥3 physicians treated an OHCA case (nighttime duty), *n* (%)	8	(66.7)
≥3 nurses treated an OHCA case (daytime duty), *n* (%)	6	(50.0)
≥3 nurses treated an OHCA case (nighttime duty), *n* (%)	2	(16.7)
Acute care physicians for OHCA treatments, *n* (%)	12	(100.0)
Intensive care physicians for OHCA treatments, *n* (%)	10	(83.3)
ICU training facility for board-certified intensivists, *n* (%)	10	(83.3)
Anesthesiologists for OHCA treatments, *n* (%)	8	(66.7)
Cardiologists for OHCA treatments, *n* (%)	11	(91.7)
Pediatricians for OHCA treatments, *n* (%)	8	(66.7)
Use of ETCO_2_ monitor during cardiopulmonary arrest, *n* (%)	8	(66.7)
ECPR use for OHCA, *n* (%)	9	(75.0)
ECPR protocol, *n* (%)	9	(75.0)
Clinical engineer who performed ECPR priming, *n* (%)	10	(83.3)
Body temperature management for OHCA, *n* (%)	12	(100)
Body temperature management protocol, *n* (%)	10	(83.3)
Target (maintenance) temperature (°C), *n* (%)^a^		
33 °C	2	(20.0)
34 °C	7	(70.0)
35 °C	1	(10.0)
Duration of target (maintenance) temperature (hours), mean (SD)^a^	24	(0.0)
Rewarming target temperature (°C), mean (SD)^a^	36.1	(0.3)
Duration of rewarming (hours), mean (SD)^a^	33.6	(20.3)

*OHCA* out-of-hospital cardiac arrest, *ICU* intensive care unit, *ETCO*_*2*_ end-tidal carbon dioxide, *ECPR* extracorporeal cardiopulmonary resuscitation, *SD* standard deviation

^a^Calculated for ten institutions having body temperature management protocol

Table 2 shows baseline characteristics of 657 OHCA patients. The average age was 66.2 years old, and 15 were children aged <17 years old. Males accounted for 66.2 % of patients. The proportion of the cardiac cause of OHCAs was 50.4 %. A total of 187 (28.5 %) patients had ROSC after hospital arrival and 55 (8.4 %) patients had already received ROSC by the time of their arrival. Among these patients, 21 (3.2 %) had VF/pulseless ventricular tachycardia (VT), 148 (22.5 %) had pulseless electrical activity (PEA), and 435 (66.2 %) were asystole as the first documented rhythm after hospital arrival.

**Table 2:** Baseline characteristics

	Total	
	(*n* = 657)	
Age, year, mean (SD)	66.2	(20.2)
Children aged 0–17 years old, *n* (%)	15	(2.3)
Male, *n* (%)	406	(61.8)
Cause, *n* (%)		
Cardiac	331	(50.4)
Acute coronary syndrome	159	(24.2)
Other heart disease	21	(3.2)
Presumed cardiac cause	151	(23.0)
Non-cardiac	326	(49.6)
Cerebrovascular disease	18	(2.7)
Respiratory disease	31	(4.7)
Malignant tumor	8	(1.2)
External	216	(32.9)
Traffic injury	38	(5.8)
Fall	54	(8.2)
Hanging	26	(4.0)
Drowning	23	(3.5)
Asphyxia	57	(8.7)
Drug overuse	5	(0.8)
Other external cause	13	(2.0)
Others	53	(8.1)
SIDS (only for children)	3	(0.5)
Departure of ambulance or helicopter with physicians, *n* (%)	107	(16.3)
ROSC status, *n* (%)		
ROSC after hospital arrival	187	(28.5)
ROSC before hospital arrival	55	(8.4)
No ROSC	415	(63.2)
First documented rhythm after hospital arrival, *n* (%)		
VF/pulseless VT	21	(3.2)
PEA	148	(22.5)
Asystole	435	(66.2)
Presence of pulse	53	(8.1)

*SD* standard deviation, *SIDS* sudden infant death syndrome, *ROSC* return of spontaneous circulation, *VF* ventricular fibrillation, *VT* ventricular fibrillation, *PEA* pulseless electrical

Prehospital characteristics based on the Utstein template are noted in Table 3. A total of 251 (38.2 %) patients were witnessed by bystanders and 334 (50.8 %) were not. Approximately one third received bystander-initiated CPR, but only three received shocks by public-access AEDs. VF/pulseless VT as first documented rhythm was 11.6 %, PEA was 23.4 %, and asystole was 54.5 %. As for prehospital treatments by EMS personnel, 15.5 % received shocks, 21.8 % intubation, 29.1 % intravenous fluid administration, and 15.2 % adrenaline administration. The mean time interval from call to CPR started by EMS at the scene was 9.8 min and from call to hospital arrival was 32.2 min.

**Table 3:** Prehospital characteristics

	Total	
	(*n* = 657)	
Witness status, *n* (%)		
Witnessed by bystanders	251	(38.2)
Family member	135	(20.5)
Friend	11	(1.7)
Colleague	9	(1.4)
Passerby	33	(5.0)
Others	63	(9.6)
Witnessed by EMS personnel	72	(11.0)
Not witnessed	334	(50.8)
Bystander-initiated CPR, *n* (%)		
Yes	210	(31.9)
Chest compression—only CPR	167	(25.4)
Conventional CPR with rescue breathing	43	(6.5)
No	447	(68.1)
Shock by public-access AEDs, *n* (%)	3	(0.5)
Dispatcher instructions, *n* (%)	243	(37.0)
First documented rhythm, *n* (%)		
VF/pulseless VT	77	(11.6)
PEA	154	(23.4)
Asystole	358	(54.5)
Others	68	(10.4)
Shock by EMS personnel, *n* (%)	102	(15.5)
Advanced airway management, *n* (%)		
None	309	(47.0)
Esophageal obturator airway	195	(29.7)
Endotracheal intubation	143	(21.8)
Laryngeal mask airway	10	(1.5)
Intravenous fluid, *n* (%)	191	(29.1)
Adrenaline administration, *n* (%)	100	(15.2)
Call to CPR started by EMS, min, mean (SD)	9.8	(6.0)
Call to hospital arrival, min, mean (SD)	32.2	(9.9)

*EMS* emergency medical service, *CPR* cardiopulmonary resuscitation, *VF* ventricular fibrillation, *PEA* pulseless electrical activity, *AED* automated external defibrillator, *SD* standard deviation

In-hospital data by the type of first documented rhythm at EMS arrival are noted in Table 4. After hospital arrival, 10.5 % received defibrillation, 90.8 % tracheal intubation, 3.0 % ECPR, 2.9 % IABP, 6.4 % CAG, 3.5 % PCI, and 83.1 % adrenaline administration. The proportion of implementation differed by the type of first documented rhythm. As for arterial blood gases and laboratory data measured initially at hospital arrival, the mean values were as follows: pH was 6.9, PaCO_2_ 86.1 mmHg, PaO_2_ 79.2 mmHg, base excess −16.4 mEq/l, lactate 13.2 mmol/l, creatinine 1.4 mg/dl, potassium 6.3 mEq/l, and ammonia 283.8 μg/dl. The values differed by the type of first documented rhythm.

**Table 4:** In-hospital advanced treatments, drug administrations, and arterial blood gases by the type of first documented rhythm at EMS arrival

	Total		First documented rhythm at EMS arrival						
			VF/pulseless VT		PEA/Asystole		Others		*P*
	(*n* = 657)		(*n* = 77)		(*n* = 512)		(*n* = 68)		
Defibrillation, *n* (%)	69	(10.5)	29	(37.7)	32	(6.3)	8	(11.8)	<0.001
Tracheal intubation, *n* (%)									0.013
Yes	449	(68.3)	61	(79.2)	334	(65.2)	54	(79.4)	
Intubated by EMS personnel in prehospital settings	148	(22.5)	14	(18.2)	126	(24.6)	8	(11.8)	
No	60	(9.1)	2	(2.6)	52	(10.2)	6	(8.8)	
Extracorporeal life support, *n* (%)	20	(3.0)	13	(16.9)	6	(1.2)	1	(1.5)	<0.001
Intra-aortic balloon pumping, *n* (%)	19	(2.9)	12	(15.6)	5	(1.0)	2	(2.9)	<0.001
Coronary angiography, *n* (%)	42	(6.4)	31	(40.3)	8	(1.6)	3	(4.4)	<0.001
Percutaneous coronary intervention, *n* (%)	23	(3.5)	17	(22.1)	5	(1.0)	1	(1.5)	<0.001
Target temperature management, *n* (%)	61	(9.3)	34	(44.2)	23	(4.5)	4	(5.9)	<0.001
Drug administration during cardiac arrest (multiple choice)									
Adrenaline, *n* (%)	546	(83.1)	57	(74.0)	436	(85.2)	53	(77.9)	0.021
Amiodarone, *n* (%)	10	(1.5)	9	(11.7)	1	(0.2)	0	(0.0)	<0.001
Nifekalant, *n* (%)	11	(1.7)	8	(10.4)	3	(0.6)	0	(0.0)	<0.001
Lidocaine, *n* (%)	13	(2.0)	9	(11.7)	3	(0.6)	1	(1.5)	<0.001
Atropine, *n* (%)	15	(2.3)	6	(7.8)	8	(1.6)	1	(1.5)	0.003
Magnesium, *n* (%)	13	(2.0)	9	(11.7)	3	(0.6)	1	(1.5)	<0.001
Vasopressin, *n* (%)	0	(0.0)	0	(0.0)	0	(0.0)	0	(0.0)	
Arterial blood gases at hospital arrival, mean (SD)^a^									
pH	6.93	(0.19)	7.02	(0.18)	6.91	(0.19)	7.01	(0.19)	<0.001
PaCO_2_ (mmHg)	86.1	(37.8)	69.8	(30.6)	91.0	(38.2)	70.1	(33.1)	<0.001
PaO_2_ (mmHg)	79.3	(107.3)	126.0	(138.9)	71.6	(98.6)	79.7	(112.8)	<0.001
HCO_3_ (mEq/l)	16.1	(5.6)	16.4	(4.8)	16.2	(5.7)	15.1	(5.3)	0.342
Base excess (mEq/l)	-16.4	(7.5)	-14.6	(7.8)	-16.8	(7.5)	-15.2	(6.9)	0.020
Lactate (mmol/l)	13.2	(5.4)	11.1	(4.7)	13.8	(5.4)	11.9	(5.3)	<0.001
Glucose (mg/dl)	223	(126)	265	(113)	219	(125)	206	(137)	0.007
Laboratory data at hospital arrival, mean (SD)^a^									
Blood urea nitrogen (mg/dl)	25.3	(21.9)	20.6	(11.1)	24.3	(18.2)	39.8	(42.7)	<0.001
Creatinine (mg/dl)	1.4	(1.2)	1.3	(1.1)	1.4	(1.2)	1.7	(1.2)	0.139
Total protein (g/dl)	6.0	(1.0)	6.1	(1.0)	6.0	(1.0)	5.8	(1.3)	0.320
Albumin (g/dl)	3.1	(0.7)	3.3	(0.7)	3.0	(0.7)	3.0	(0.9)	0.019
Sodium (mEq/l)	139.9	(7.7)	139.7	(4.9)	139.8	(8.4)	140.7	(4.5)	0.723
Potassium (mEq/l)	6.3	(2.8)	4.5	(1.3)	6.7	(2.9)	5.6	(2.1)	<0.001
Ammonia (μg/dl)	283.8	(287)	128	(114)	325	(307)	195	(215)	0.025

*EMS* emergency medical service, *SD* standard deviation

^a^Calculated only for patients with gases or data

Table 5 shows the outcomes among 657 OHCA patients. A total of 197 patients (30 %) were admitted to an intensive care unit/ward. The proportions of 1-month survival and CPC 1/2 at 1 month after OHCAs were 9.0 % and 3.0 %, respectively. The proportions of 90-day survival and CPC 1/2 at 90 days after OHCA occurrence were 5.9 % and 3.0 %, respectively. Not all children survived during the study period.

**Table 5:** Outcomes

	Total	
	(*n* = 657)	
Condition after hospital arrival, *n* (%)		
Admitted to ICU/ward	197	(30.0)
Death at the ED	460	(70.0)
1-month survival, *n* (%)		
Yes	59	(9.0)
Hospitalized	40	(6.1)
Discharge to survival	18	(2.7)
Unknown	1	(0.2)
No	598	(91.0)
CPC 1 month after OHCAs, *n* (%)		
CPC 1	18	(2.7)
CPC 2	2	(0.3)
CPC 3	7	(1.1)
CPC 4	32	(4.9)
CPC 5	583	(88.7)
P-CPC 6 (only for children)	15	(2.3)
90-day survival, *n* (%)		
Yes	39	(5.9)
Hospitalized	12	(1.8)
Discharge to survival	26	(3.8)
Unknown	1	(0.2)
No	612	(93.2)
Unknown	6	(0.9)
CPC 90 days after OHCAs, *n* (%)		
CPC 1	19	(2.9)
CPC 2	1	(0.2)
CPC 3	2	(0.3)
CPC 4	16	(2.4)
CPC 5	597	(90.9)
Unknown	7	(1.1)
P-CPC 6	15	(2.3)

*ICU* intensive care unit, *ED* emergency department, *CPC* cerebral performance category, *OHCA* out-of-hospital cardiac arrest

## Discussion

In July 2012, we launched a multi-center, prospective observational registry (the CRITICAL study) in Osaka, Japan that focused on OHCA patients by EMS personnel who were transported to CCMCs or hospitals with an emergency care department. Herein, we described the study design and its rationale and briefly presented characteristics and outcomes of 657 OHCA patients in the first half of the year after the study’s initiation.

The CRITICAL study group established a comprehensive cohort, assessing and collecting both pre- and in-hospital data regarding OHCA patients in Osaka. In this study, we have had the following three purposes. First, we made a uniform registry form regarding the emergency system of transported institutions, as well as in-hospital procedures such as PCI, TTM, and ECPR, in order to clarify the actual situation of OHCA treatments after hospital arrival. Second, by assessing the different emergency systems in transported institutions (e.g., CCMCs or not), we could provide appropriate criteria for hospital selection by EMS in line with each patients’ characteristics such as age, gender, and the presence or absence of prehospital ROSC. In addition, our data would be of help in constructing appropriate emergency medical systems by finding factors associated with hospital selection. Third, we could produce a systematic therapeutic strategy to improve the neurological outcome of OHCA patients after hospital arrival by verifying the effectiveness of in-hospital advanced treatments such as the use of drugs, ELS, PCI, and TTM. Thus, we consider that the CRITICAL study will contribute to improving patient outcomes after OHCAs in the target area.

The CRITICAL study has several strengths. It is well-known from preceding studies that basic life supports such as chest compressions or defibrillations are more effective for improving OHCA outcomes than advanced life supports [26]. Therefore, to properly assess effects of in-hospital procedures such as PCI, TTM, and ECPR, the OHCA registry system, including in-hospital data, should be conducted in areas where prehospital emergency care systems are established adequately, as in Osaka [3–5]. In Osaka, we have a robust emergency medical network with EMS personnel, physicians, and researchers, functioning as the Osaka Utstein Project since 1998 [3–5, 27]. In addition, Osaka, with about 9 million inhabitants of all ages, has both urban and rural areas and findings from the CRITICAL study that could be applied to other communities in Japan as well as worldwide.

Our preliminary data demonstrated that the prehospital VF/pulseless VT group was more likely to receive advanced in-hospital procedures such as ECPR, IABP, CAG, PCI, and drug administrations than the other rhythm groups. The CPR guidelines recommend that comatose cardiac arrest survivors receive TTM after VF/pulseless VT [1, 28], but the present study showed that only 44.2 % of the prehospital VF/pulseless VT group received TTM. This result suggested that it would take much time to implement changes in CPR guidelines into emergency care systems, even in CCMCs [29, 30]. In addition, a detailed method of TTM implementation in actual settings, such as an optimal temperature, timing of introduction, cooling duration, and cooling method [31], and other PCAS treatments such as ECPR [15, 32] and PCI [11] was reported to improve the outcome after OHCAs but has not yet been established. Importantly, the appropriate of usage of these treatment strategies is under debate [33]. By collecting a large number of OHCA patients, our registry could solve these problems in the future.

Most importantly, the survival from OHCAs is very low and there is room for improvement worldwide. In this study, the proportions of 1-month survival and 1-month survival with favorable neurological outcome defined as CPC 1 or 2 [23, 24] after OHCAs were 9.0 % and 3.0 %, respectively. These results were almost the same as in our previous studies [5, 34] and another report [35]. According to AHA consensus statements, longer-term end points such as 90-day neurocognitive function and quality-of-life assessments after cardiac arrests should be considered [25]. This is because OHCA patients’ neurological condition might fluctuate during the first 90 days after arrests. Therefore, survival and the CPC at 90 days after OHCAs would provide a reasonable outcome parameter for large-scale OHCA cohorts like ours. The CRITICAL study was designed to obtain survival and CPC at 90 days after OHCA occurrence based on this recommendation. However, there could be a potential loss of patient long-term follow-up. Seven cases (1.1 %) in this study could not be followed-up in the first half year of observation. Hence, we must obtain all available long-term data regarding outcomes among OHCA patients with ROSC and enhance the accuracy of our study because registered OHCA patients in our database will greatly increase.

Because medical resources are limited, we should provide medical institutions with appropriate transportation criteria. Further evidence gathered from evaluating the effect of advanced treatments should also be accumulated in order to build a uniform protocol that can be used in all hospitals including CCMCs. The CRITICAL study will be suitable for these purposes.

The CRITICAL study has some inherent limitations, however. First, it is a hospital-based observation and cannot follow all OHCA patients in the target area. We are calling for other medical institutions in Osaka to participate in our project and to include OHCA patients in this area as much as possible. Second, we were able to assess survival and neurologic status at 90 days after OHCAs, but much longer follow-ups (e.g., outcomes at 1 year after arrests) are not available. Finally, unmeasured confounding factors might have affected the relationship between measured factors and the outcomes after OHCAs.

## Conclusions

We launched the CRITICAL study in July 2012, and this ongoing study continues to gather participants. This registry enrolls over 2000 OHCA patients every year and is ongoing without a set conclusion to the study period, in order to provide valuable information regarding appropriate therapeutic strategies for OHCA patients.

## Abbreviations

CAG: cardioangiography
CCMC: critical care medical center
CPC: cerebral performance category
CPR: cardiopulmonary resuscitation
ECPR: extracorporeal cardiopulmonary resuscitation
EMS: emergency medical service
IABP: intra-aortic balloon pumping
OHCA: out-of-hospital cardiac arrest
PCAS: post-cardiac arrest syndrome
PCI: percutaneous coronary intervention
PEA: pulseless electrical activity
ROSC: return of spontaneous circulation
TTM: target temperature management
VF: ventricular fibrillation
VT: ventricular tachycardia
